# Measurement of acetabular wall indices: comparison between CT and plain radiography

**DOI:** 10.1093/jhps/hnab008

**Published:** 2021-07-19

**Authors:** Jaron Nazaroff, Bryan Mark, James Learned, Dean Wang

**Affiliations:** 1 University of California Irvine School of Medicine, 1001 Health Sciences Rd, Irvine, CA 92617, USA; 2 Department of Orthopaedic Surgery, University of California Irvine Health, 101 The City Drive South, Pavilion III, Building 29A, Orange, CA 92868, USA

## Abstract

The purpose of this study was to compare measurements of anterior wall index (AWI) and posterior wall index (PWI) on computed tomography (CT) to those on radiographs (XR). A consecutive cohort of 33 patients (45 hips total) being evaluated for hip pain with both XR and CT was examined. Preoperative measurements of AWI and PWI were performed utilizing supine anteroposterior pelvic XR and coronal and swiss axial CT scans by two independent raters. Mean differences between XR and CT measurements were compared, and agreement between measurements was assessed using the concordance correlation coefficient (*r_c_*) and Bland–Altman analysis. A total of 39 hips in 28 patients were analyzed. The mean patient age was 31.1 ± 9.0 years, and 50% were female. Mean AWI and PWI on XR was 0.50 ± 0.14 and 0.91 ± 0.12, respectively. Measured values of AWI were consistently larger (0.08 ± 0.10, *P* < 0.01) on XR compared with both coronal and swiss axial CT, with moderate agreement between XR and CT measurements (*r_c_* = 0.68–0.70). Measured values of PWI were consistently smaller (0.15 ± 0.12, *P* < 0.05) on XR compared with both coronal and swiss axial CT, with poor agreement between XR and CT measurements (*r_c_* = 0.37–0.45). Measured values of acetabular wall indices on XR were consistently larger for AWI and smaller for PWI relative to CT. Agreement between XR and CT measures of the indices were moderate to poor. This highlights the need for standardization of XR- and CT-based measurements to improve assessment of acetabular coverage and subsequent clinical decision-making.

## INTRODUCTION

Various measures of acetabular coverage have been described and are routinely evaluated clinically on plain radiographs (XR) [1–5]. Siebenrock *et al*. [6] first described measuring the relative acetabular coverage contributed by the anterior wall and posterior wall using the anterior wall index (AWI) and posterior wall index (PWI), respectively. The advantage of such indices is that the measurements can be performed on a single anteroposterior (AP) pelvic XR and are applicable regardless of femoral head and patient size. 

However, the ability to fully evaluate acetabular overcoverage, undercoverage or version is limited on plain radiography alone [7–9]. These measurements are susceptible to subtle changes in rotational alignment of the pelvis during XR acquisition as well as beam divergence, potentially leading to measurement error [10]. Due to the limitations of plain films, there has been increased utilization of computed tomography (CT) for improved three-dimensional characterization of bony morphology of the hip [7, 8, 11–14]. Currently, many clinicians routinely use CT to more accurately quantify the three-dimensional projections of acetabular coverage for preoperative planning. However, some have noted that identical measures on XR and CT are not always in agreement [15–18]. Chadayammuri *et al*. [15] compared XR measurements of the lateral center-edge angle (LCEA) to those on CT and found them to be consistently larger on CT. Additionally, measures of acetabular component version in total hip arthroplasty patients can often be discordant between XR and CT [16, 18]. Although good correlation between acetabular wall indices and a validated computer model calculating percentage of anterior and posterior acetabular coverage from XR has been reported [6], whether measurement of acetabular wall indices on XR are concordant with those measured on CT is unknown. Measurement of AWI and PWI on XR would be advantageous over CT due to lower cost and radiation dose for these younger patients with hip pain. Therefore, the purpose of this study was to evaluate the mean difference and degree of agreement between measurements of acetabular wall indices on XR versus CT.

## MATERIALS AND METHODS

**Fig. 1.**
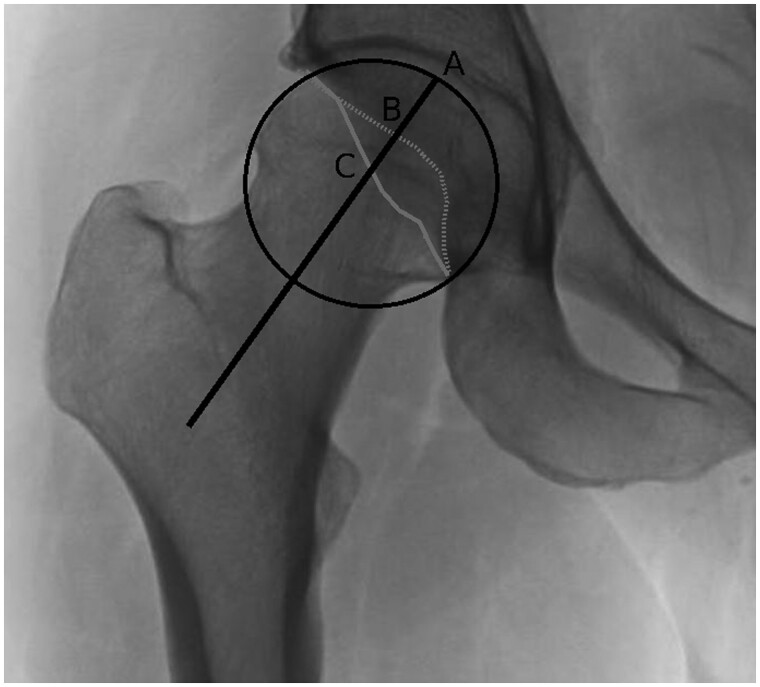
Identification and measurement of anterior and posterior anterior wall coverage on XR. A best fit circle is placed to encompass the femoral head. The anterior acetabular wall is identified with the dashed line. The posterior acetabular wall is identified with the solid line. The solid black line bisects the femoral head. Anterior wall coverage is measured from the top of the femoral head (A) to intersection of the anterior acetabular wall and the bisecting femoral head line (B). Posterior wall coverage is measured from the top of the femoral head (A) to the intersection of the posterior acetabular wall and the bisecting femoral head line (C).

After obtaining institutional review board approval (HS# 2019-5175), data were collected prospectively from a consecutive cohort of 33 patients (45 hips) who were seen in the senior author’s practice at a single academic institution for hip pain from 2018 to 2020 and received concurrent XR and CT imaging during their evaluation. Patients who were >50 years of age and had only prior outside CT imaging were excluded from this study. Additionally, hips having Tonnis grade ≥2 or history of prior surgery were excluded from this study. Preoperative measurements of AWI and PWI were performed by two independent raters blinded to the treatment outcomes. Indices were performed on supine AP pelvic XR and both coronal and swiss axial (axial oblique) CT scans [19]. LCEA in the manner described by Ogata *et al*. [20] and anterior center-edge angle (ACEA) were measured on supine AP pelvis and false profile XR, respectively. AWI and PWI were measured on XR in the manner described by Siebenrock *et al*. [6]. Briefly, AWI and PWI were calculated by measuring anterior and posterior acetabular wall coverage, respectively, and dividing those distances by the radius of the femoral head (Fig. 1).

**Fig. 2.**
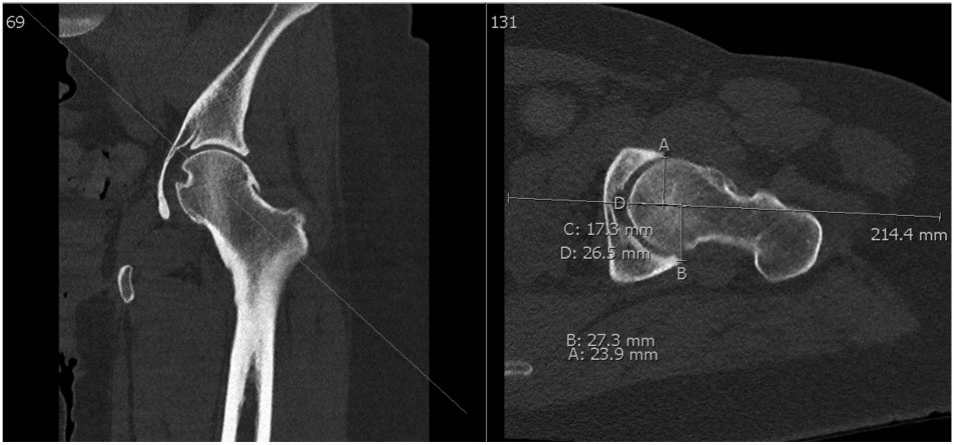
CT coronal and CT swiss axial cross sections imaging demonstrating methodology for calculating the AWI and PWI coverages. A reference line is placed through the center of the femoral head. The distance from the femoral head along the reference line to line A represents the AW coverage. This is repeat to line B for posterior wall coverage. This is represented by lines C and D in the image below (anterior wall coverage and posterior wall coverage, respectively). Line D is on top of line C. To calculate the AWI/PWI the AW and PW is divided by the radius of the femoral head.

Three-dimensional CT was performed at one facility per protocol described by Heyworth *et al*. [11] and was routinely obtained by the senior author for preoperative planning. For measurements of AWI and PWI on the coronal series, coronal and swiss axial images were placed side by side, and a mid-coronal location on the coronal series was determined by a corresponding scout line transecting the center of femoral head on swiss axial series. To measure the radius of the femoral head, a circle was drawn over the center of femoral head that best encompassed the femoral head shape and center of rotation on both the coronal and swiss axial projections. Two radius measurements were made and then averaged. AWI and PWI measurements were then obtained by drawing a fixed reference line in the plane of the swiss axial through the center of the femoral head along the axis of the neck as well as a fixed reference point at the intersection of this line and the most medial aspect of the femoral head. While scrolling anteriorly through the slices of the coronal series, visualization of the anterior wall was confirmed using the scout line on swiss axial view. The distance from the fixed reference point on the medial femoral head to the anterior wall along the reference line was measured. This measurement was then divided by the radius to give the AWI. The PWI was similarly measured by scrolling posteriorly through the slices of the coronal series to the posterior wall (Fig. 2, Supplementary Appendix A).

Measurements of AWI and PWI on the swiss axial series were done in a similar fashion. Coronal and swiss axial images were placed side by side, the mid-swiss axial location on the swiss axial series was determined by a corresponding scout line transecting the center of femoral head on coronal series. On that single slice, reference lines orthogonal to the horizontal plane were made adjacent to the anterior wall and posterior wall. The distance from the most medial aspect of the femoral head to the reference lines in the horizontal plane were measured. These measurements were then divided by the radius of the femoral head to calculate the AWI and PWI (Fig. 2, Supplementary Appendix A).

Inter-rater reliability was assessed using the intraclass correlation coefficient (ICC). Normal distribution was confirmed using the Kolmogorov–Smirnov test. Mean differences between XR and CT measurements and over/undermeasurement proportions were assessed using the paired *t*-test and chi-squared test, respectively. Agreement between XR and CT measurements was assessed using the concordance correlation coefficient (*r_c_*) and Bland–Altman limits of agreement analysis. The *r_c_* was used to assess agreement between XR and CT measurements since it does not assume an underlying analysis of variance model and is commonly used to evaluate agreement between diagnostic tests [21]. However, in most scenarios, the *r_c_* is very similar to the ICC [22]. Correlation coefficients of >0.75 is considered excellent, 0.75–0.40 is considered fair and <0.40 is considered poor [23]. Power analysis indicated that a minimum of 28 paired XR and CT studies were needed to test for a mean index difference of 0.1 with a power of 80% and alpha set to 0.05. Data are reported as mean ± standard deviation for measurements in each group. All statistical analyses were performed using Microsoft Excel (Redmond, WA, USA) and GraphPad Prism 8 (San Diego, CA, USA).

## RESULTS

Six pairs of XR and CT studies were excluded due to >50 years of age or outside CT imaging, resulting in 39 hips in 28 patients for analysis. Of this cohort, the mean patient age was 31.1 ± 9.0 years, and 50% were female (Table I). On XR, mean LCEA and ACEA were 30.3 ± 8.5° and 31.0 ± 10.0°, respectively. The majority of patients (71%) had normal LCEA as defined by 25–40°, and 12% of patients had an LCEA less than 20°. Mean AWI and PWI measured on XR were 0.50 ± 0.14 and 0.91 ± 0.12, respectively.

**Table I.** Patient demographics and baseline characteristics

Patient variables	Data
Total number of patients (no. of hips), *N*	28 (39)
Female gender, *n* (%)	14 (50)
Age (years), mean (SD)	31.1 (9)
BMI (kg/m^2^), mean (SD)	27.5 (5.32)
LCEA (°), mean (SD)	30.3° (8.5°)
ACEA (°), mean (SD)	31.0° (10.1°)

ACEA, anterior center edge angle; BMI, body mass index; LCEA, lateral center edge angle; SD, standard deviation.

Inter-rater reliability for acetabular wall indices on XR, coronal CT and swiss axial CT imaging were excellent for the AWI and PWI (Table II). The ICC for AWI measurements was 0.92 on XR, 0.92 on coronal CT and 0.92 on swiss axial CT. The ICC for PWI measurements was 0.90 on XR, 0.85 on coronal CT and 0.78 on swiss axial CT (Table II).

**Table II.** Inter-rater agreement for AWI and PWI

Wall indices	Imaging modality	ICC	95% CI
AWI			
	XR	0.924	0.861–0.959
	CT coronal	0.915	0.845–0.955
	CT swiss	0.918	0.849–0.956
PWI			
	XR	0.903	0.822–0.948
	CT coronal	0.851	0.728–0.92
	CT swiss	0.786	0.558–0.893

AWI, anterior wall index; CI, confidence interval; ICC, intraclass correlation coefficient; PWI, posterior wall index; XR, plain radiograph.

**Fig. 3.**
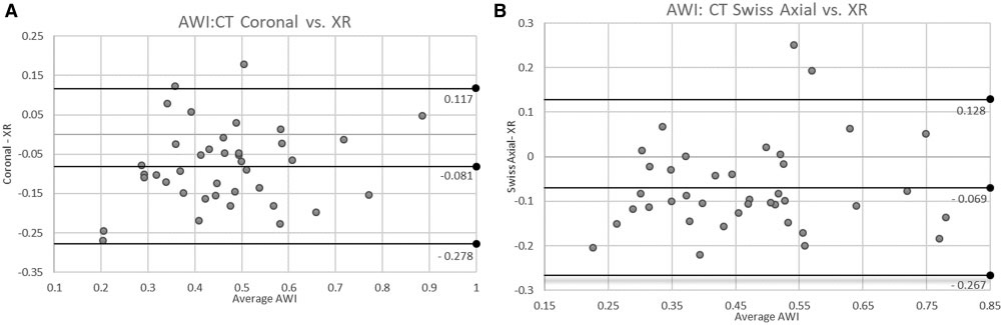
Bland–Altman plots evaluating AWI CT coronal and CT swiss axial versus XR measurements. (**A** and **B**) Bland–Altman plots depicting variability of measurements of wall indices on plain radiography versus CT. (**A**) AWI: CT coronal versus XR. (**B**) AWI: CT swiss axial versus XR plotting the data reveals AWI XR measurements that were greater than both CT modalities.

**Fig. 4.**
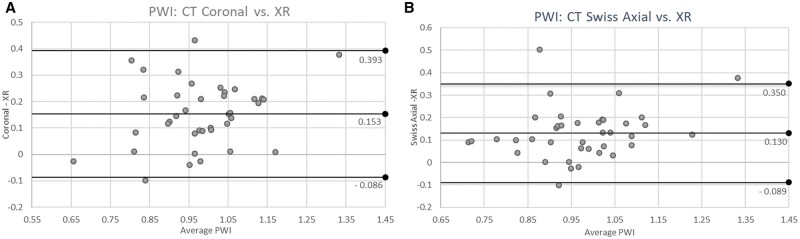
Bland–Altman plots evaluating PWI CT coronal and CT swiss axial versus XR measurements. (**A** and **B**) Bland–Altman plots depicting variability of measurements of wall indices on plain radiography versus CT: (**A**) PWI: CT coronal versus XR. (**B**) PWI: CT swiss axial versus XR plots (**A**) and (**B**) reveal PWI XR measurements that were less than both CT modalities.

Mean AWI and PWI measured on coronal CT were 0.42 ± 0.16 and 1.06 ± 0.15, respectively. Mean AWI and PWI measured on swiss axial CT were 0.43 ± 0.16 and 1.04 ± 0.15, respectively. Measured values of AWI were larger on XR compared with both coronal CT (mean difference of 0.08 ± 0.10; *P* < 0.01) and swiss axial CT (mean difference of 0.07 ± 0.10; *P* < 0.01). Bland–Altman plots revealed AWI XR measurements were generally greater than both CT coronal [−0.081 (95% limit of agreement (LOA) −0.278 to 0.117)] and CT swiss axial measurements [−0.069 (95% LOA −0.267 to 0.128)] (Fig. 3). In contrast, measured values of PWI were smaller on XR compared with both coronal CT (mean difference of 0.15 ± 0.12; *P* < 0.01) and swiss axial CT (mean difference of 0.13 ± 0.11; *P* < 0.01). Bland–Altman plots revealed PWI XR measurements were generally less than both CT coronal [0.153 (95% LOA −0.086 to 0.393)] and CT swiss axial measurements [0.130 (95% LOA −0.089 to 0.350)] (Fig. 4).

For correlation analysis between XR and CT modalities, moderate agreement was observed between XR and CT measurements of AWI (*r_c_* = 0.68 and 0.70 for coronal and swiss axial, respectively). Poor agreement was observed between XR and CT measurements of PWI (*r_c_* = 0.37 and 0.45 for coronal and swiss axial, respectively) (Table III). Excellent agreement was observed between coronal and swiss axial CT indices measurements (*r_c_* = 0.79 for AWI, 0.88 for PWI).

**Table III.** Modality comparison

Wall index	Comparison	*r_c_*	95% CI	Mean difference (CT-XR)	*T*-test *P*-value	Chi-squared *P*-value
AWI						
	CT Cor versus XR	0.685	0.507–0.807	−0.081	<0.01	<0.01
	CT Swiss versus XR	0.699	0.518–0.819	−0.069	<0.01	<0.01
PWI						
	CT Cor versus XR	0.369	0.187–0.527	0.154	<0.01	<0.01
	CT Swiss versus XR	0.447	0.254–0.604	0.13	<0.01	<0.01

AWI, anterior wall index; CI, confidence interval; Cor, coronal; CT, computed tomography; PWI, posterior wall index; *r_c_*, concordance correlation coefficient; Swiss, swiss axial; XR, plain radiograph.

## DISCUSSION

The AWI and PWI are clinically used to quantify anterior and posterior wall acetabular coverage, respectively, using AP pelvic XR. However, these measurements are susceptible to subtle changes in rotational alignment of the pelvis and XR projection error. Due to these limitations, there is increased attention on the role of CT imaging to evaluate acetabular morphology and femoral head coverage [7, 8, 11, 13, 24, 25]. The purpose of this study was to evaluate the mean difference and degree of agreement between measurements of acetabular wall indices on XR versus CT.

Inter-rater reliability assessed using the ICC for measurements of XR, CT coronal and CT swiss axial imaging was overall excellent (0.79–0.92) for the AWI and PWI. The ICC results for the XR PWI and AWI measurements were similar to the results reported by Siebenrock *et al*. [6]. This is of particular importance to the findings of the current study as notable differences in measured values of AWI and PWI between XR and CT were found. As consistent with findings reported in the literature, measurements of anatomic pelvic parameters among XR and CT can often be discrepant with one another [15–19]. In a prospective cohort study, Chadayammuri *et al*. [15] found that the LCEA was on average 2.1° larger on CT compared with XR (32.9° versus 30.8°). Furthermore, the authors found even greater disparities between CT and XR existed in patients with acetabular dysplasia and femoroacetabular impingement (FAI). Wylie *et al*. [26] further investigated this inconsistency in LCEA measurements between XR and CT and found that the sourcil-edge LCEA represents the anterosuperior acetabular coverage while the bone-edge LCEA represents superior/lateral coverage, indicating the shortcomings with measurement of a three-dimensional anatomic feature on a two-dimensional XR projection. In contrast to LCEA or ACEA, this study examined a ratio measure of acetabular coverage, which may not be as sensitive to these AP variations since linear measurements are being normalized to the radius of the femoral head in each respective study type. Additionally, whereas LCEA measurement was limited to the lateral edge on a single CT slice in the Chadaymmuri *et al*. study, we did not have such limitations on coronal CT because scrolling through the scan was necessary to identify the edges of the anterior wall and posterior wall in the swiss axial plane.

In the current study, variable agreement ranging from poor to excellent (*r_c_* = 0.37–0.88) was observed across XR and CT comparisons (Table III). In a group of patients with hip pain, Siebenrock *et al*. [6] found good correlation between acetabular wall indices measured on XR and a validated computer model that calculates the percentage of anterior and posterior acetabular coverage from an AP pelvis XR. Validation of this computer model, Hip^2^Norm, involved a comparison of XR with CT data [27]. However, to our knowledge, direct comparison of acetabular wall indices measurements on XR and CT studies has not been performed previously. When performing this direct comparison, we found statistically significant differences between measurement of AWI and PWI on XR versus CT. Whether these differences are clinically significant is unclear since normal ranges of these parameters have not been well-defined [6, 28]. Compared to the means reported in a group of normal asymptomatic adults (0.35 for AWI and 1.12 for PWI [28]), the mean differences of 0.08 and 0.15 indicate a 23% and 13% change for AWI and PWI, respectively. These differences may be large enough to change the classification of patients from normal to dysplastic or normal to overcovered and vice versa. For instance, a difference of 0.08 on the AWI at the lower end of values could result in a clinician deciding to do a periacetabular osteotomy or at the higher end of values could determine whether the patient has a pincer lesion that can be treated with acetabuloplasty.

Although we did not classify patients based on hip-specific etiology (dysplasia, normal, overcoverage, retroversion, etc.), we found that measured values of acetabular wall indices on XR were consistently larger for AWI and smaller for PWI relative to both coronal and swiss axial CT. Since both XR and CT were obtained with the patient in the supine position, we don’t believe position-dependent changes in pelvic tilt contributed significantly to these discrepancies. Nevertheless, compared to CT, XR may be more sensitive to subtle pelvic tilting and rotation, which are difficult to limit during XR capture. Additionally, radiograph projection due to beam divergence from a central source may have also accounted for the discrepancies found in this study, contributing to the moderate and poor agreement between XR and CT for AWI and PWI, respectively. This projection error is stronger in the lateral parts of an XR image and can be approximately 6° for the hip on an AP pelvis XR [3, 29]. Similarly, XR projection focused over the hip, rather than a true AP XR will further alter the appearance of the acetabular rim, enlarging the anterior rim through its proximity to the source [30]. Such projection error could in theory result in a falsely elevated AWI on XR.

There are several limitations to this study. Some of the larger differences seen in the Bland–Altman plots (Figs 3 and 4) may have been attributed to deficiencies in the acetabular indices themselves. Measurements of AWI and PWI assumes a perfectly spherical femoral head. In normal and particularly in non-spherical femoral heads, such as those with CAM lesions, the projected radius is subject to change depending on the CT slice and axial rotation of the hip during imaging. Additionally, on some AP films, demarcation of the anterior or posterior wall was difficult due to the body habitus of the patient. This highlights the deficiency of this method when measuring on XR. 

Another limitation involves measurement on the swiss axial scans, which were reformatted based on determination of the long axis of the femoral neck and therefore subject to off-axis error. While not a CT-based study, Stelzeneder *et al*. [29] demonstrated that slice selection on two-dimensional magnetic resonance imaging influenced measurement of acetabular coverage, and CT may also be sensitive to changes in measurement depending on the slices analyzed. Several patients had calcifications of the anterosuperior labrum often seen in FAI. Although the raters specifically avoided these calcifications during measurement, these calcifications were more notable on CT compared to XR and could lead to overestimation of anterior wall coverage on CT.

Lastly, all patients in this study were being treated for symptomatic hip pain. Therefore, the findings of this study may not be applicable to all populations and should be validated in a cohort of asymptomatic individuals.

In summary, overall measured values of acetabular wall indices on XR were consistently larger for AWI and smaller for PWI relative to CT. Agreement between XR and CT measures of the indices were moderate to poor. These discrepancies highlight the need for standardization and validation of XR- and CT-based measurements to improve assessment of acetabular coverage in order to inform clinical decision-making. Further investigation to compare radiographic measurements of acetabular coverage with three-dimensional volumetric measures calculated from CT are warranted.

## DATA AVAILABILITY STATEMENT

The data underlying this article will be shared on reasonable request to the corresponding author.

## SUPPLEMENTARY DATA

Supplementary data are available at *Journal of Hip Preservation Surgery* online.

## CONFLICT OF INTEREST STATEMENT

None declared. 

## Supplementary Material

hnab008_Supplementary_Data
