# Supplementary material for: Measurement of acetabular wall indices: comparison between CT and plain radiography
Source: J Hip Preserv Surg. 2021 Jul 19;8(1):51–7. doi: 10.1093/jhps/hnab008 (PMC8460168; doi:10.1093/jhps/hnab008)
Supplement: hnab008_Supplementary_Data [file hnab008_supplementary_data.docx]

**Appendix A.** AWI/ PWI Measurements on CT

Coronal Measurements:

1. Coronal and swiss axial CT images placed adjacent to one another.
2. Find mid cross sections of the femoral head on both coronal and swiss axial views.
3. When controlling the swiss axial view, a line representing the swiss axial plane will appear on the coronal view. The top end of that line will be used as the anchor. The anchor is adjacent to the blue star in the image below. (Appendix Image 1)
4. Measure the distance from the anchor to the femoral head along the swiss axial plane line. This is the reference distance. (Appendix Image 2)
5. Calculate the radius by measuring the diameter of the femoral head. Take two measurements and average them.
6. While controlling the coronal view, find the anterior wall. The coronal plane line will overlay the anterior wall on the swiss axial view. (Appendix Image 3)
7. Click on the swiss axial view, the swiss axial plane line will appear on the coronal view. Measure the distance from the anchor to the farthest edge of the anterior wall along the swiss axial plane line (this is done on the coronal view). This distance minus the reference distance is the anterior wall coverage. (Appendix Image 4)
8. Anterior wall coverage/radius = AWI.
9. While controlling the coronal view, find the posterior wall. The coronal plane line will overlay the posterior wall on the swiss axial view. (Appendix Image 5)
10. Click on the swiss axial view, the swiss axial plane line will appear on the coronal view. Measure the distance from the anchor to the farthest edge of the posterior wall along the swiss axial plane line. This distance minus the reference distance is the posterior wall coverage. (Appendix Image 6)
11. Posterior wall coverage/radius = PWI.

Swiss Axial Measurements:

1. Coronal and swiss axial CT images placed adjacent to one another.
2. Find mid cross sections of the femoral head on both views.
3. Click on the coronal view. The coronal plane line will appear on the swiss axial view. Draw a line that overlays the coronal plane line on the swiss axial view. The distance does not matter. This line is the reference line. (Appendix Image 7)
4. Draw a perpendicular line from the tip of the anterior wall to the reference line made in step 3 (represented by line A). Repeat for the posterior wall (represented by line B). Distances do not matter. (Appendix Image 8)
5. Measure the distance from the femoral head along the reference line to line A. This is anterior wall coverage. Repeat to line B for posterior wall coverage. This is represented by lines C and D in the image below (anterior wall coverage and posterior wall coverage, respectively). Line D is on top of line C. (Appendix Image 9)
6. Calculate the radius by measuring the diameter of the femoral head. Take two measurements and average them.
7. Anterior wall coverage/ radius= AWI
8. Posterior wall coverage/ radius= PWI
